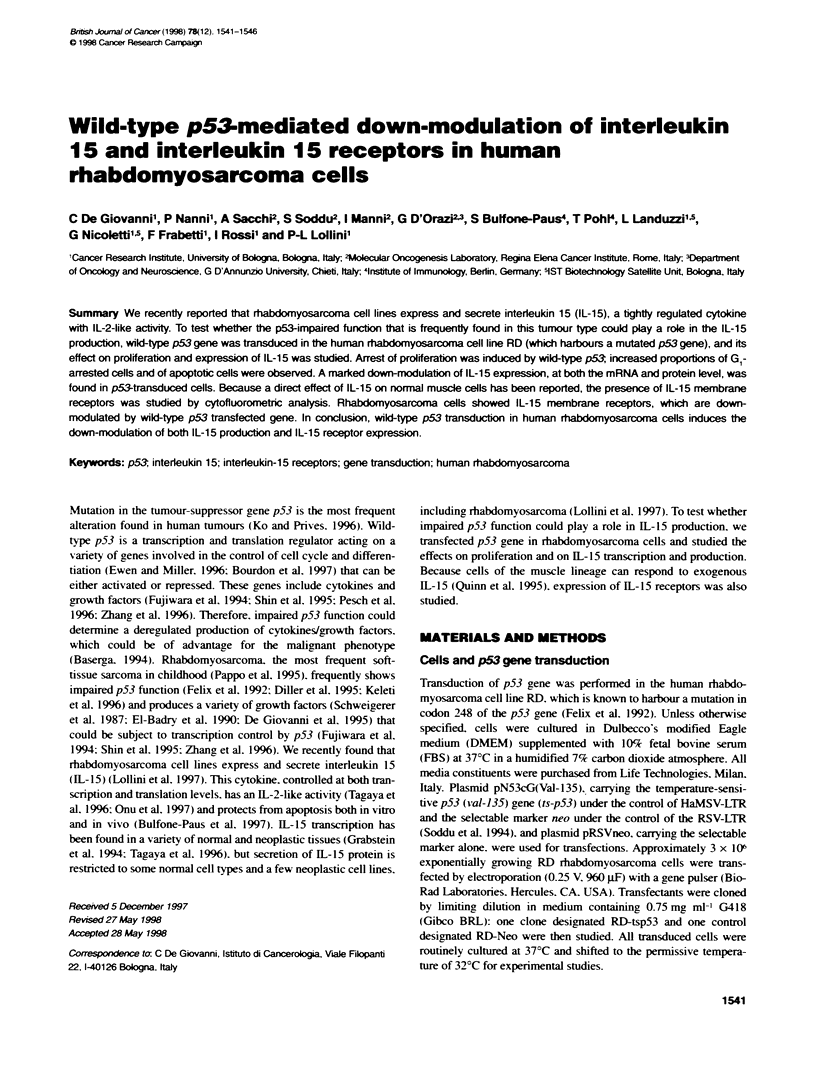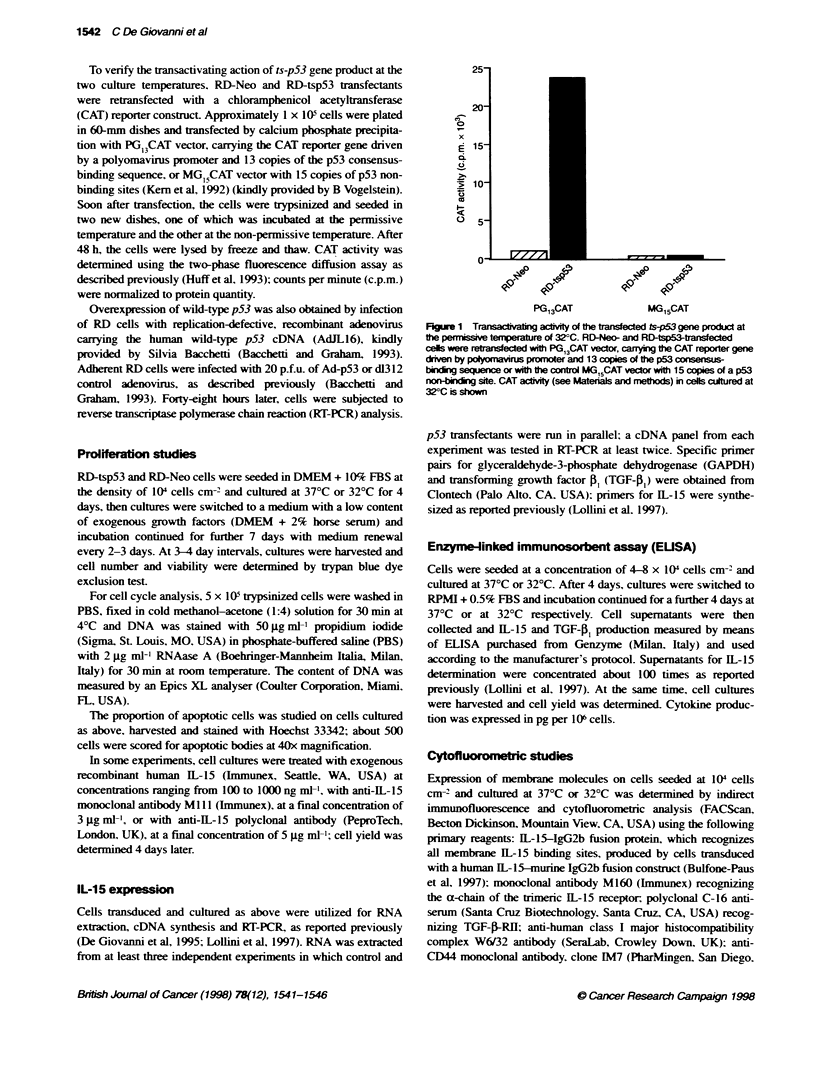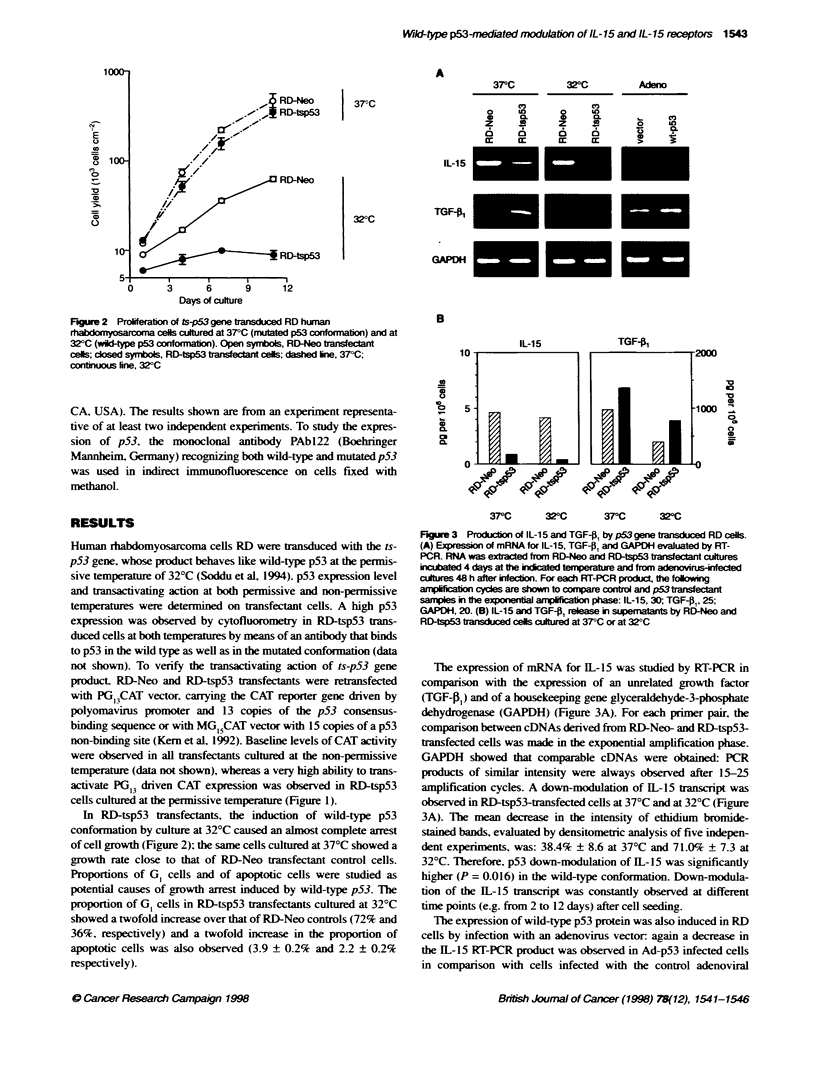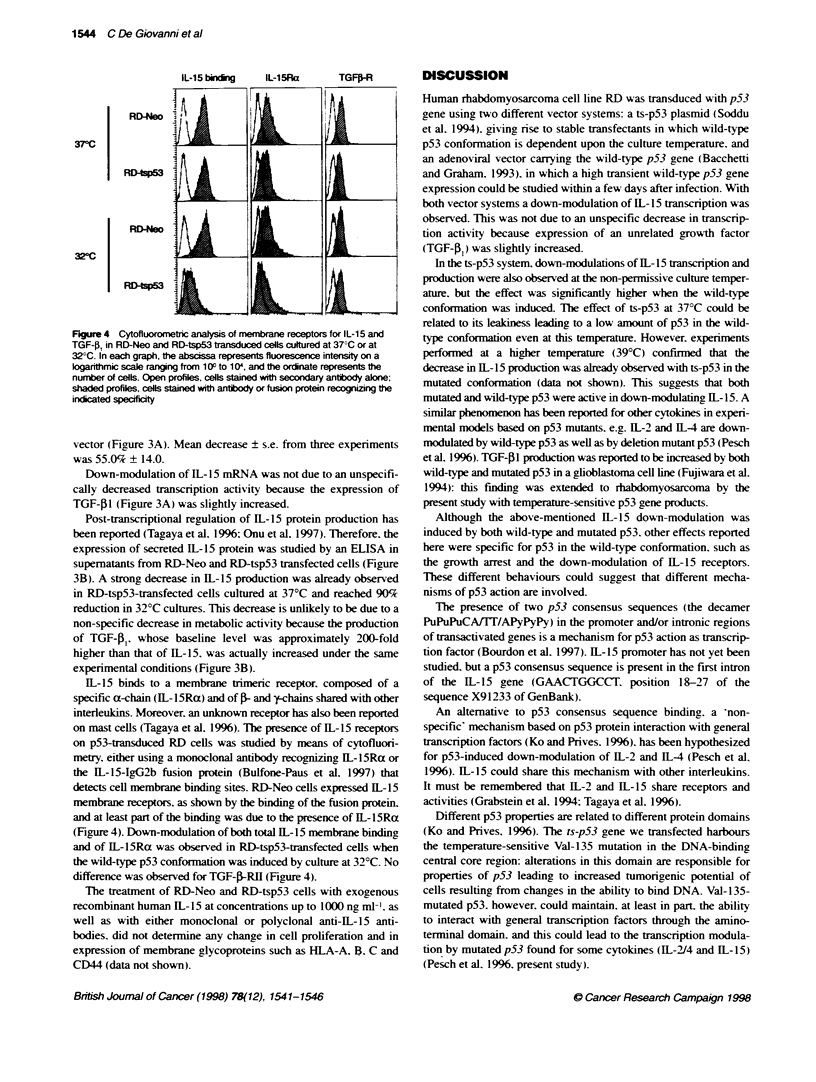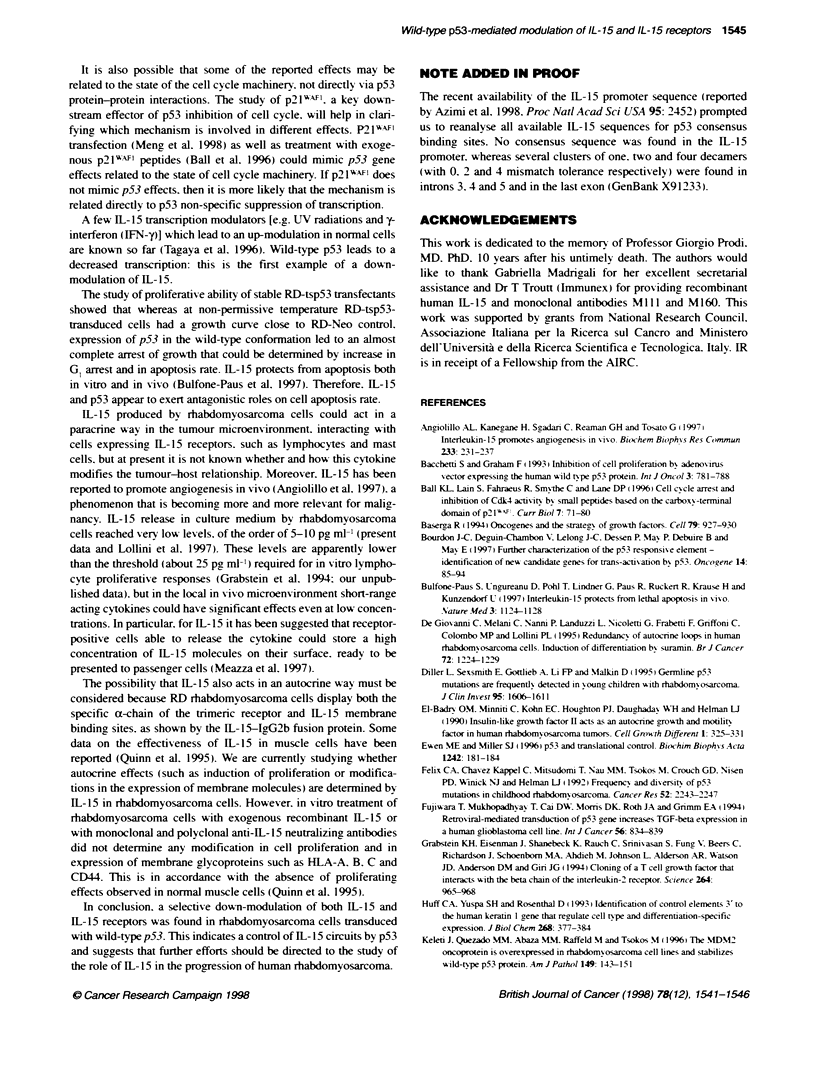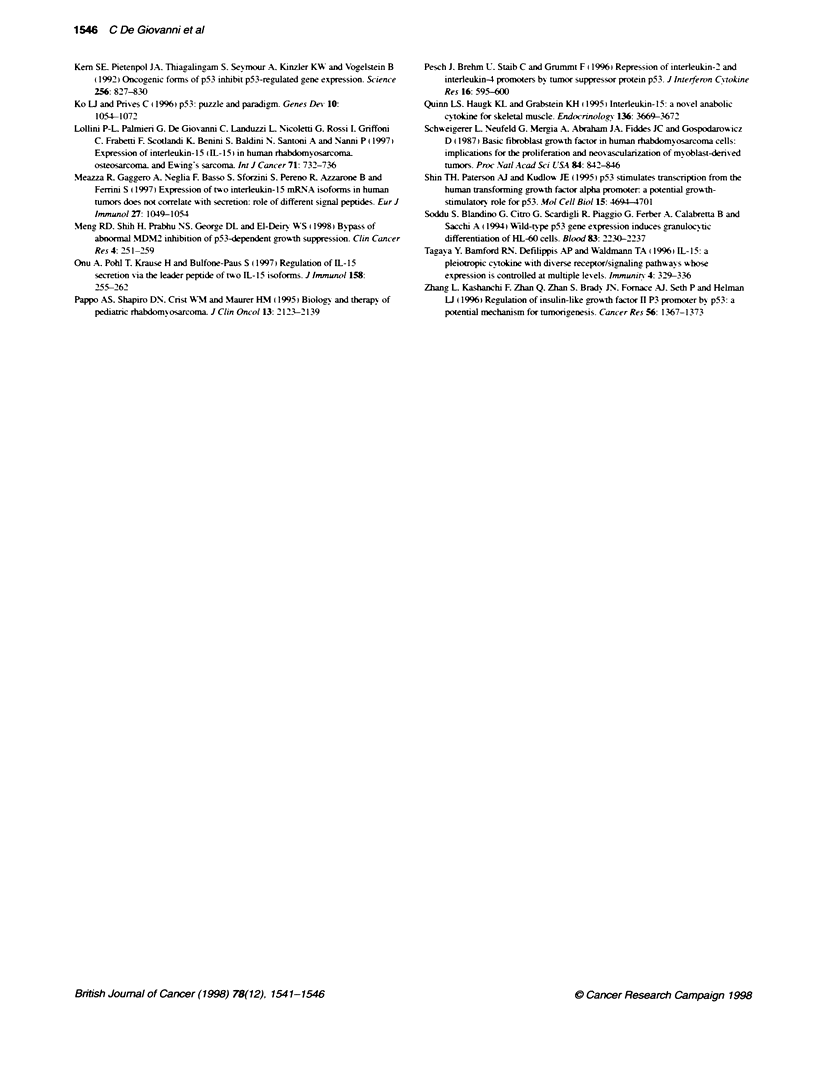# Wild-type p53-mediated down-modulation of interleukin 15 and interleukin 15 receptors in human rhabdomyosarcoma cells.

**DOI:** 10.1038/bjc.1998.721

**Published:** 1998-12

**Authors:** C. De Giovanni, P. Nanni, A. Sacchi, S. Soddu, I. Manni, G. D'Orazi, S. Bulfone-Paus, T. Pohl, L. Landuzzi, G. Nicoletti, F. Frabetti, I. Rossi, P. L. Lollini

**Affiliations:** Cancer Research Institute, University of Bologna, Italy.

## Abstract

**Images:**


British Journal of Cancer (1998) 78(12). 1541–1546
© 1998 Cancer Research Campaign

### Wild-type p53-mediated down-modulation of interleukin 15 and interleukin 15 receptors in human rhabdomyosarcoma cells

**C De Giovanni[1], P Nanni[1], A Sacchi[2], S Soddu[2], I Manni[2], G D'Orazi[2,3], S Bulfone-Paus[4], T Pohl[4], L Landuzzi[1,5], G Nicoletti[1,5], F Frabetti[1], I Rossi[1] and P-L Lollini[1]**

[1]Cancer Research Institute, University of Bologna, Bologna, Italy; [2]Molecular Oncogenesis Laboratory, Regina Elena Cancer Institute, Rome, Italy; [3]Department of Oncology and Neuroscience, G D'Annunzio University, Chieti, Italy; [4]Institute of Immunology, Berlin, Germany; [5]IST Biotechnology Satellite Unit, Bologna, Italy

**Summary** We recently reported that rhabdomyosarcoma cell lines express and secrete interleukin 15 (IL-15), a tightly regulated cytokine with IL-2-like activity. To test whether the p53-impaired function that is frequently found in this tumour type could play a role in the IL-15 production, wild-type *p53* gene was transduced in the human rhabdomyosarcoma cell line RD (which harbours a mutated *p53* gene), and its effect on proliferation and expression of IL-15 was studied. Arrest of proliferation was induced by wild-type *p53*; increased proportions of $G_1$-arrested cells and of apoptotic cells were observed. A marked down-modulation of IL-15 expression, at both the mRNA and protein level, was found in *p53*-transduced cells. Because a direct effect of IL-15 on normal muscle cells has been reported, the presence of IL-15 membrane receptors was studied by cytofluorometric analysis. Rhabdomyosarcoma cells showed IL-15 membrane receptors, which are down-modulated by wild-type *p53* transfected gene. In conclusion, wild-type *p53* transduction in human rhabdomyosarcoma cells induces the down-modulation of both IL-15 production and IL-15 receptor expression.

**Keywords:** *p53*; interleukin 15; interleukin-15 receptors; gene transduction; human rhabdomyosarcoma

Mutation in the tumour-suppressor gene *p53* is the most frequent alteration found in human tumours (Ko and Prives, 1996). Wild-type *p53* is a transcription and translation regulator acting on a variety of genes involved in the control of cell cycle and differentiation (Ewen and Miller, 1996; Bourdon et al, 1997) that can be either activated or repressed. These genes include cytokines and growth factors (Fujiwara et al, 1994; Shin et al, 1995; Pesch et al, 1996; Zhang et al, 1996). Therefore, impaired *p53* function could determine a deregulated production of cytokines/growth factors, which could be of advantage for the malignant phenotype (Baserga, 1994). Rhabdomyosarcoma, the most frequent soft-tissue sarcoma in childhood (Pappo et al, 1995), frequently shows impaired *p53* function (Felix et al, 1992; Diller et al, 1995; Keleti et al, 1996) and produces a variety of growth factors (Schweigerer et al, 1987; El-Badry et al, 1990; De Giovanni et al, 1995) that could be subject to transcription control by *p53* (Fujiwara et al, 1994; Shin et al, 1995; Zhang et al, 1996). We recently found that rhabdomyosarcoma cell lines express and secrete interleukin 15 (IL-15) (Lollini et al, 1997). This cytokine, controlled at both transcription and translation levels, has an IL-2-like activity (Tagaya et al, 1996; Onu et al, 1997) and protects from apoptosis both in vitro and in vivo (Bulfone-Paus et al, 1997). IL-15 transcription has been found in a variety of normal and neoplastic tissues (Grabstein et al, 1994; Tagaya et al, 1996), but secretion of IL-15 protein is restricted to some normal cell types and a few neoplastic cell lines, including rhabdomyosarcoma (Lollini et al, 1997). To test whether impaired *p53* function could play a role in IL-15 production, we transfected *p53* gene in rhabdomyosarcoma cells and studied the effects on proliferation and on IL-15 transcription and production. Because cells of the muscle lineage can respond to exogenous IL-15 (Quinn et al, 1995), expression of IL-15 receptors was also studied.

*Received 5 December 1997*
*Revised 27 May 1998*
*Accepted 28 May 1998*

*Correspondence to:* C De Giovanni, Istituto di Cancerologia, Viale Filopanti 22, I-40126 Bologna, Italy

### MATERIALS AND METHODS

### Cells and *p53* gene transduction

Transduction of *p53* gene was performed in the human rhabdomyosarcoma cell line RD, which is known to harbour a mutation in codon 248 of the *p53* gene (Felix et al, 1992). Unless otherwise specified, cells were cultured in Dulbecco's modified Eagle medium (DMEM) supplemented with 10% fetal bovine serum (FBS) at 37°C in a humidified 7% carbon dioxide atmosphere. All media constituents were purchased from Life Technologies, Milan, Italy. Plasmid pN53cG(Val-135), carrying the temperature-sensitive *p53* (*val-135*) gene (*ts-p53*) under the control of HaMSV-LTR and the selectable marker *neo* under the control of the RSV-LTR (Soddu et al, 1994), and plasmid pRSVneo, carrying the selectable marker alone, were used for transfections. Approximately $3 \times 10^6$ exponentially growing RD rhabdomyosarcoma cells were transfected by electroporation (0.25 V, 960 μF) with a gene pulser (Bio-Rad Laboratories, Hercules, CA, USA). Transfectants were cloned by limiting dilution in medium containing 0.75 mg $ml^{-1}$ G418 (Gibco BRL): one clone designated RD-tsp53 and one control designated RD-Neo were then studied. All transduced cells were routinely cultured at 37°C and shifted to the permissive temperature of 32°C for experimental studies.

To verify the transactivating action of *ts-p53* gene product at the two culture temperatures, RD-Neo and RD-tsp53 transfectants were retransfected with a chloramphenicol acetyltransferase (CAT) reporter construct. Approximately $1 \times 10^5$ cells were plated in 60-mm dishes and transfected by calcium phosphate precipitation with $PG_{13}CAT$ vector, carrying the CAT reporter gene driven by a polyomavirus promoter and 13 copies of the p53 consensus-binding sequence, or $MG_{15}CAT$ vector with 15 copies of p53 non-binding sites (Kern et al, 1992) (kindly provided by B Vogelstein). Soon after transfection, the cells were trypsinized and seeded in two new dishes, one of which was incubated at the permissive temperature and the other at the non-permissive temperature. After 48 h, the cells were lysed by freeze and thaw. CAT activity was determined using the two-phase fluorescence diffusion assay as described previously (Huff et al, 1993); counts per minute (c.p.m.) were normalized to protein quantity.

Overexpression of wild-type *p53* was also obtained by infection of RD cells with replication-defective, recombinant adenovirus carrying the human wild-type *p53* cDNA (AdJL16), kindly provided by Silvia Bacchetti (Bacchetti and Graham, 1993). Adherent RD cells were infected with 20 p.f.u. of Ad-p53 or dl312 control adenovirus, as described previously (Bacchetti and Graham, 1993). Forty-eight hours later, cells were subjected to reverse transcriptase polymerase chain reaction (RT-PCR) analysis.

**Figure 1** Transactivating activity of the transfected *ts-p53* gene product at the permissive temperature of 32°C. RD-Neo- and RD-tsp53-transfected cells were retransfected with $PG_{13}CAT$ vector, carrying the CAT reporter gene driven by polyomavirus promoter and 13 copies of the p53 consensus-binding sequence or with the control $MG_{15}CAT$ vector with 15 copies of a p53 non-binding site. CAT activity (see Materials and methods) in cells cultured at 32°C is shown

### Proliferation studies

RD-tsp53 and RD-Neo cells were seeded in DMEM + 10% FBS at the density of $10^4$ cells $cm^{-2}$ and cultured at 37°C or 32°C for 4 days, then cultures were switched to a medium with a low content of exogenous growth factors (DMEM + 2% horse serum) and incubation continued for further 7 days with medium renewal every 2–3 days. At 3–4 day intervals, cultures were harvested and cell number and viability were determined by trypan blue dye exclusion test.

For cell cycle analysis, $5 \times 10^5$ trypsinized cells were washed in PBS, fixed in cold methanol–acetone (1:4) solution for 30 min at 4°C and DNA was stained with 50 µg $ml^{-1}$ propidium iodide (Sigma, St. Louis, MO, USA) in phosphate-buffered saline (PBS) with 2 µg $ml^{-1}$ RNAase A (Boehringer-Mannheim Italia, Milan, Italy) for 30 min at room temperature. The content of DNA was measured by an Epics XL analyser (Coulter Corporation, Miami, FL, USA).

The proportion of apoptotic cells was studied on cells cultured as above, harvested and stained with Hoechst 33342; about 500 cells were scored for apoptotic bodies at 40× magnification.

In some experiments, cell cultures were treated with exogenous recombinant human IL-15 (Immunex, Seattle, WA, USA) at concentrations ranging from 100 to 1000 ng $ml^{-1}$, with anti-IL-15 monoclonal antibody M111 (Immunex), at a final concentration of 3 µg $ml^{-1}$, or with anti-IL-15 polyclonal antibody (PeproTech, London, UK), at a final concentration of 5 µg $ml^{-1}$; cell yield was determined 4 days later.

### IL-15 expression

Cells transduced and cultured as above were utilized for RNA extraction, cDNA synthesis and RT-PCR, as reported previously (De Giovanni et al, 1995; Lollini et al, 1997). RNA was extracted from at least three independent experiments in which control and *p53* transfectants were run in parallel; a cDNA panel from each experiment was tested in RT-PCR at least twice. Specific primer pairs for glyceraldehyde-3-phosphate dehydrogenase (GAPDH) and transforming growth factor $\beta_1$ (TGF-$\beta_1$) were obtained from Clontech (Palo Alto, CA, USA); primers for IL-15 were synthesized as reported previously (Lollini et al, 1997).

### Enzyme-linked immunosorbent assay (ELISA)

Cells were seeded at a concentration of 4–8 × $10^4$ cells $cm^{-2}$ and cultured at 37°C or 32°C. After 4 days, cultures were switched to RPMI + 0.5% FBS and incubation continued for a further 4 days at 37°C or at 32°C respectively. Cell supernatants were then collected and IL-15 and TGF-$\beta_1$ production measured by means of ELISA purchased from Genzyme (Milan, Italy) and used according to the manufacturer's protocol. Supernatants for IL-15 determination were concentrated about 100 times as reported previously (Lollini et al, 1997). At the same time, cell cultures were harvested and cell yield was determined. Cytokine production was expressed in pg per $10^6$ cells.

### Cytofluorometric studies

Expression of membrane molecules on cells seeded at $10^4$ cells $cm^{-2}$ and cultured at 37°C or 32°C was determined by indirect immunofluorescence and cytofluorometric analysis (FACScan, Becton Dickinson, Mountain View, CA, USA) using the following primary reagents: IL-15–IgG2b fusion protein, which recognizes all membrane IL-15 binding sites, produced by cells transduced with a human IL-15–murine IgG2b fusion construct (Bulfone-Paus et al, 1997); monoclonal antibody M160 (Immunex) recognizing the α-chain of the trimeric IL-15 receptor; polyclonal C-16 antiserum (Santa Cruz Biotechnology, Santa Cruz, CA, USA) recognizing TGF-β-RII; anti-human class I major histocompatibility complex W6/32 antibody (SeraLab, Crowley Down, UK); anti-CD44 monoclonal antibody, clone IM7 (PharMingen, San Diego,

© Cancer Research Campaign 1998

**Figure 2** Proliferation of *ts-p53* gene transduced RD human rhabdomyosarcoma cells cultured at 37°C (mutated p53 conformation) and at 32°C (wild-type p53 conformation). Open symbols, RD-Neo transfectant cells; closed symbols, RD-tsp53 transfectant cells; dashed line, 37°C; continuous line, 32°C

CA, USA). The results shown are from an experiment representative of at least two independent experiments. To study the expression of *p53*, the monoclonal antibody PAb122 (Boehringer Mannheim, Germany) recognizing both wild-type and mutated *p53* was used in indirect immunofluorescence on cells fixed with methanol.

### RESULTS

Human rhabdomyosarcoma cells RD were transduced with the *ts-p53* gene, whose product behaves like wild-type p53 at the permissive temperature of 32°C (Soddu et al, 1994). p53 expression level and transactivating action at both permissive and non-permissive temperatures were determined on transfectant cells. A high p53 expression was observed by cytofluorometry in RD-tsp53 transduced cells at both temperatures by means of an antibody that binds to p53 in the wild type as well as in the mutated conformation (data not shown). To verify the transactivating action of *ts-p53* gene product, RD-Neo and RD-tsp53 transfectants were retransfected with $PG_{13}$CAT vector, carrying the CAT reporter gene driven by polyomavirus promoter and 13 copies of the *p53* consensus-binding sequence or with $MG_{15}$CAT vector with 15 copies of a p53 non-binding site (Kern et al, 1992). Baseline levels of CAT activity were observed in all transfectants cultured at the non-permissive temperature (data not shown), whereas a very high ability to transactivate $PG_{13}$ driven CAT expression was observed in RD-tsp53 cells cultured at the permissive temperature (Figure 1).

In RD-tsp53 transfectants, the induction of wild-type p53 conformation by culture at 32°C caused an almost complete arrest of cell growth (Figure 2); the same cells cultured at 37°C showed a growth rate close to that of RD-Neo transfectant control cells. Proportions of $G_1$ cells and of apoptotic cells were studied as potential causes of growth arrest induced by wild-type *p53*. The proportion of $G_1$ cells in RD-tsp53 transfectants cultured at 32°C showed a twofold increase over that of RD-Neo controls (72% and 36%, respectively) and a twofold increase in the proportion of apoptotic cells was also observed (3.9 ± 0.2% and 2.2 ± 0.2% respectively).

**Figure 3** Production of IL-15 and TGF-$\beta_1$ by *p53* gene transduced RD cells. (**A**) Expression of mRNA for IL-15, TGF-$\beta_1$ and GAPDH evaluated by RT-PCR. RNA was extracted from RD-Neo and RD-tsp53 transfectant cultures incubated 4 days at the indicated temperature and from adenovirus-infected cultures 48 h after infection. For each RT-PCR product, the following amplification cycles are shown to compare control and *p53* transfectant samples in the exponential amplification phase: IL-15, 30; TGF-$\beta_1$, 25; GAPDH, 20. (**B**) IL-15 and TGF-$\beta_1$ release in supernatants by RD-Neo and RD-tsp53 transduced cells cultured at 37°C or at 32°C

The expression of mRNA for IL-15 was studied by RT-PCR in comparison with the expression of an unrelated growth factor (TGF-$\beta_1$) and of a housekeeping gene glyceraldehyde-3-phosphate dehydrogenase (GAPDH) (Figure 3A). For each primer pair, the comparison between cDNAs derived from RD-Neo- and RD-tsp53-transfected cells was made in the exponential amplification phase. GAPDH showed that comparable cDNAs were obtained: PCR products of similar intensity were always observed after 15–25 amplification cycles. A down-modulation of IL-15 transcript was observed in RD-tsp53-transfected cells at 37°C and at 32°C (Figure 3A). The mean decrease in the intensity of ethidium bromide-stained bands, evaluated by densitometric analysis of five independent experiments, was: 38.4% ± 8.6 at 37°C and 71.0% ± 7.3 at 32°C. Therefore, p53 down-modulation of IL-15 was significantly higher ($P = 0.016$) in the wild-type conformation. Down-modulation of the IL-15 transcript was constantly observed at different time points (e.g. from 2 to 12 days) after cell seeding.

The expression of wild-type p53 protein was also induced in RD cells by infection with an adenovirus vector: again a decrease in the IL-15 RT-PCR product was observed in Ad-p53 infected cells in comparison with cells infected with the control adenoviral

**Figure 4** Cytofluorometric analysis of membrane receptors for IL-15 and TGF-$\beta_1$ in RD-Neo and RD-tsp53 transduced cells cultured at 37°C or at 32°C. In each graph, the abscissa represents fluorescence intensity on a logarithmic scale ranging from $10^0$ to $10^4$, and the ordinate represents the number of cells. Open profiles, cells stained with secondary antibody alone; shaded profiles, cells stained with antibody or fusion protein recognizing the indicated specificity vector (Figure 3A). Mean decrease ± s.e. from three experiments was 55.0% ± 14.0.

Down-modulation of IL-15 mRNA was not due to an unspecifically decreased transcription activity because the expression of TGF-β1 (Figure 3A) was slightly increased.

Post-transcriptional regulation of IL-15 protein production has been reported (Tagaya et al. 1996; Onu et al. 1997). Therefore, the expression of secreted IL-15 protein was studied by an ELISA in supernatants from RD-Neo and RD-tsp53 transfected cells (Figure 3B). A strong decrease in IL-15 production was already observed in RD-tsp53-transfected cells cultured at 37°C and reached 90% reduction in 32°C cultures. This decrease is unlikely to be due to a non-specific decrease in metabolic activity because the production of TGF-$\beta_1$, whose baseline level was approximately 200-fold higher than that of IL-15, was actually increased under the same experimental conditions (Figure 3B).

IL-15 binds to a membrane trimeric receptor, composed of a specific α-chain (IL-15Rα) and of β- and γ-chains shared with other interleukins. Moreover, an unknown receptor has also been reported on mast cells (Tagaya et al. 1996). The presence of IL-15 receptors on p53-transduced RD cells was studied by means of cytofluorimetry, either using a monoclonal antibody recognizing IL-15Rα or the IL-15-IgG2b fusion protein (Bulfone-Paus et al. 1997) that detects cell membrane binding sites. RD-Neo cells expressed IL-15 membrane receptors, as shown by the binding of the fusion protein, and at least part of the binding was due to the presence of IL-15Rα (Figure 4). Down-modulation of both total IL-15 membrane binding and of IL-15Rα was observed in RD-tsp53-transfected cells when the wild-type p53 conformation was induced by culture at 32°C. No difference was observed for TGF-β-RII (Figure 4).

The treatment of RD-Neo and RD-tsp53 cells with exogenous recombinant human IL-15 at concentrations up to 1000 ng $ml^{-1}$, as well as with either monoclonal or polyclonal anti-IL-15 antibodies, did not determine any change in cell proliferation and in expression of membrane glycoproteins such as HLA-A, B, C and CD44 (data not shown).

### DISCUSSION

Human rhabdomyosarcoma cell line RD was transduced with *p53* gene using two different vector systems: a ts-p53 plasmid (Soddu et al. 1994), giving rise to stable transfectants in which wild-type p53 conformation is dependent upon the culture temperature, and an adenoviral vector carrying the wild-type *p53* gene (Bacchetti and Graham, 1993), in which a high transient wild-type *p53* gene expression could be studied within a few days after infection. With both vector systems a down-modulation of IL-15 transcription was observed. This was not due to an unspecific decrease in transcription activity because expression of an unrelated growth factor (TGF-$\beta_1$) was slightly increased.

In the ts-p53 system, down-modulations of IL-15 transcription and production were also observed at the non-permissive culture temperature, but the effect was significantly higher when the wild-type conformation was induced. The effect of ts-p53 at 37°C could be related to its leakiness leading to a low amount of p53 in the wild-type conformation even at this temperature. However, experiments performed at a higher temperature (39°C) confirmed that the decrease in IL-15 production was already observed with ts-p53 in the mutated conformation (data not shown). This suggests that both mutated and wild-type p53 were active in down-modulating IL-15. A similar phenomenon has been reported for other cytokines in experimental models based on p53 mutants, e.g. IL-2 and IL-4 are down-modulated by wild-type p53 as well as by deletion mutant p53 (Pesch et al. 1996). TGF-β1 production was reported to be increased by both wild-type and mutated p53 in a glioblastoma cell line (Fujiwara et al. 1994): this finding was extended to rhabdomyosarcoma by the present study with temperature-sensitive p53 gene products.

Although the above-mentioned IL-15 down-modulation was induced by both wild-type and mutated p53, other effects reported here were specific for p53 in the wild-type conformation, such as the growth arrest and the down-modulation of IL-15 receptors. These different behaviours could suggest that different mechanisms of p53 action are involved.

The presence of two *p53* consensus sequences (the decamer PuPuPuCA/TT/APyPyPy) in the promoter and/or intronic regions of transactivated genes is a mechanism for p53 action as transcription factor (Bourdon et al. 1997). IL-15 promoter has not yet been studied, but a p53 consensus sequence is present in the first intron of the IL-15 gene (GAACTGGCCT, position 18–27 of the sequence X91233 of GenBank).

An alternative to p53 consensus sequence binding, a 'non-specific' mechanism based on p53 protein interaction with general transcription factors (Ko and Prives, 1996), has been hypothesized for p53-induced down-modulation of IL-2 and IL-4 (Pesch et al. 1996). IL-15 could share this mechanism with other interleukins. It must be remembered that IL-2 and IL-15 share receptors and activities (Grabstein et al. 1994; Tagaya et al. 1996).

Different p53 properties are related to different protein domains (Ko and Prives, 1996). The *ts-p53* gene we transfected harbours the temperature-sensitive Val-135 mutation in the DNA-binding central core region: alterations in this domain are responsible for properties of *p53* leading to increased tumorigenic potential of cells resulting from changes in the ability to bind DNA. Val-135-mutated p53, however, could maintain, at least in part, the ability to interact with general transcription factors through the amino-terminal domain, and this could lead to the transcription modulation by mutated *p53* found for some cytokines (IL-2/4 and IL-15) (Pesch et al. 1996, present study).

© Cancer Research Campaign 1998

It is also possible that some of the reported effects may be related to the state of the cell cycle machinery, not directly via p53 protein–protein interactions. The study of p21[WAF1], a key downstream effector of p53 inhibition of cell cycle, will help in clarifying which mechanism is involved in different effects. P21[WAF1] transfection (Meng et al, 1998) as well as treatment with exogenous p21[WAF1] peptides (Ball et al, 1996) could mimic *p53* gene effects related to the state of cell cycle machinery. If p21[WAF1] does not mimic *p53* effects, then it is more likely that the mechanism is related directly to p53 non-specific suppression of transcription.

A few IL-15 transcription modulators [e.g. UV radiations and γ-interferon (IFN-γ)] which lead to an up-modulation in normal cells are known so far (Tagaya et al, 1996). Wild-type p53 leads to a decreased transcription: this is the first example of a down-modulation of IL-15.

The study of proliferative ability of stable RD-tsp53 transfectants showed that whereas at non-permissive temperature RD-tsp53-transduced cells had a growth curve close to RD-Neo control, expression of *p53* in the wild-type conformation led to an almost complete arrest of growth that could be determined by increase in $G_1$ arrest and in apoptosis rate. IL-15 protects from apoptosis both in vitro and in vivo (Bulfone-Paus et al, 1997). Therefore, IL-15 and p53 appear to exert antagonistic roles on cell apoptosis rate.

IL-15 produced by rhabdomyosarcoma cells could act in a paracrine way in the tumour microenvironment, interacting with cells expressing IL-15 receptors, such as lymphocytes and mast cells, but at present it is not known whether and how this cytokine modifies the tumour–host relationship. Moreover, IL-15 has been reported to promote angiogenesis in vivo (Angiolillo et al, 1997), a phenomenon that is becoming more and more relevant for malignancy. IL-15 release in culture medium by rhabdomyosarcoma cells reached very low levels, of the order of 5–10 pg $ml^{-1}$ (present data and Lollini et al, 1997). These levels are apparently lower than the threshold (about 25 pg $ml^{-1}$) required for in vitro lymphocyte proliferative responses (Grabstein et al, 1994; our unpublished data), but in the local in vivo microenvironment short-range acting cytokines could have significant effects even at low concentrations. In particular, for IL-15 it has been suggested that receptor-positive cells able to release the cytokine could store a high concentration of IL-15 molecules on their surface, ready to be presented to passenger cells (Meazza et al, 1997).

The possibility that IL-15 also acts in an autocrine way must be considered because RD rhabdomyosarcoma cells display both the specific α-chain of the trimeric receptor and IL-15 membrane binding sites, as shown by the IL-15–IgG2b fusion protein. Some data on the effectiveness of IL-15 in muscle cells have been reported (Quinn et al, 1995). We are currently studying whether autocrine effects (such as induction of proliferation or modifications in the expression of membrane molecules) are determined by IL-15 in rhabdomyosarcoma cells. However, in vitro treatment of rhabdomyosarcoma cells with exogenous recombinant IL-15 or with monoclonal and polyclonal anti-IL-15 neutralizing antibodies did not determine any modification in cell proliferation and in expression of membrane glycoproteins such as HLA-A, B, C and CD44. This is in accordance with the absence of proliferating effects observed in normal muscle cells (Quinn et al, 1995).

In conclusion, a selective down-modulation of both IL-15 and IL-15 receptors was found in rhabdomyosarcoma cells transduced with wild-type *p53*. This indicates a control of IL-15 circuits by p53 and suggests that further efforts should be directed to the study of the role of IL-15 in the progression of human rhabdomyosarcoma.

### NOTE ADDED IN PROOF

The recent availability of the IL-15 promoter sequence (reported by Azimi et al, 1998, *Proc Natl Acad Sci USA* **95**: 2452) prompted us to reanalyse all available IL-15 sequences for p53 consensus binding sites. No consensus sequence was found in the IL-15 promoter, whereas several clusters of one, two and four decamers (with 0, 2 and 4 mismatch tolerance respectively) were found in introns 3, 4 and 5 and in the last exon (GenBank X91233).

### ACKNOWLEDGEMENTS

This work is dedicated to the memory of Professor Giorgio Prodi, MD, PhD, 10 years after his untimely death. The authors would like to thank Gabriella Madrigali for her excellent secretarial assistance and Dr T Troutt (Immunex) for providing recombinant human IL-15 and monoclonal antibodies M111 and M160. This work was supported by grants from National Research Council, Associazione Italiana per la Ricerca sul Cancro and Ministero dell'Università e della Ricerca Scientifica e Tecnologica, Italy. IR is in receipt of a Fellowship from the AIRC.

### REFERENCES

Angiolillo AL, Kanegane H, Sgadari C, Reaman GH and Tosato G (1997) Interleukin-15 promotes angiogenesis in vivo. *Biochem Biophys Res Commun* **233**: 231–237

Bacchetti S and Graham F (1993) Inhibition of cell proliferation by adenovirus vector expressing the human wild type p53 protein. *Int J Oncol* **3**: 781–788

Ball KL, Lain S, Fahraeus R, Smythe C and Lane DP (1996) Cell cycle arrest and inhibition of Cdk4 activity by small peptides based on the carboxy-terminal domain of p21[WAF1]. *Curr Biol* **7**: 71–80

Baserga R (1994) Oncogenes and the strategy of growth factors. *Cell* **79**: 927–930

Bourdon J-C, Deguin-Chambon V, Lelong J-C, Dessen P, May P, Debuire B and May E (1997) Further characterization of the p53 responsive element – identification of new candidate genes for trans-activation by p53. *Oncogene* **14**: 85–94

Bulfone-Paus S, Ungureanu D, Pohl T, Lindner G, Paus R, Ruckert R, Krause H and Kunzendorf U (1997) Interleukin-15 protects from lethal apoptosis in vivo. *Nature Med* **3**: 1124–1128

De Giovanni C, Melani C, Nanni P, Landuzzi L, Nicoletti G, Frabetti F, Griffoni C, Colombo MP and Lollini PL (1995) Redundancy of autocrine loops in human rhabdomyosarcoma cells. Induction of differentiation by suramin. *Br J Cancer* **72**: 1224–1229

Diller L, Sexsmith E, Gottlieb A, Li FP and Malkin D (1995) Germline p53 mutations are frequently detected in young children with rhabdomyosarcoma. *J Clin Invest* **95**: 1606–1611

El-Badry OM, Minniti C, Kohn EC, Houghton PJ, Daughaday WH and Helman LJ (1990) Insulin-like growth factor II acts as an autocrine growth and motility factor in human rhabdomyosarcoma tumors. *Cell Growth Different* **1**: 325–331

Ewen ME and Miller SJ (1996) p53 and translational control. *Biochim Biophys Acta* **1242**: 181–184

Felix CA, Chavez Kappel C, Mitsudomi T, Nau MM, Tsokos M, Crouch GD, Nisen PD, Winick NJ and Helman LJ (1992) Frequency and diversity of p53 mutations in childhood rhabdomyosarcoma. *Cancer Res* **52**: 2243–2247

Fujiwara T, Mukhopadhyay T, Cai DW, Morris DK, Roth JA and Grimm EA (1994) Retroviral-mediated transduction of p53 gene increases TGF-beta expression in a human glioblastoma cell line. *Int J Cancer* **56**: 834–839

Grabstein KH, Eisenman J, Shanebeck K, Rauch C, Srinivasan S, Fung V, Beers C, Richardson J, Schoenborn MA, Ahdieh M, Johnson L, Alderson AR, Watson JD, Anderson DM and Giri JG (1994) Cloning of a T cell growth factor that interacts with the beta chain of the interleukin-2 receptor. *Science* **264**: 965–968

Huff CA, Yuspa SH and Rosenthal D (1993) Identification of control elements 3′ to the human keratin 1 gene that regulate cell type and differentiation-specific expression. *J Biol Chem* **268**: 377–384

Keleti J, Quezado MM, Abaza MM, Raffeld M and Tsokos M (1996) The MDM2 oncoprotein is overexpressed in rhabdomyosarcoma cell lines and stabilizes wild-type p53 protein. *Am J Pathol* **149**: 143–151

© Cancer Research Campaign 1998

Kern SE. Pietenpol JA. Thiagalingam S. Seymour A. Kinzler KW and Vogelstein B (1992) Oncogenic forms of p53 inhibit p53-regulated gene expression. *Science* **256**: 827–830

Ko LJ and Prives C (1996) p53: puzzle and paradigm. *Genes Dev* **10**: 1054–1072

Lollini P-L. Palmieri G. De Giovanni C. Landuzzi L. Nicoletti G. Rossi I. Griffoni C. Frabetti F. Scotlandi K. Benini S. Baldini N. Santoni A and Nanni P (1997) Expression of interleukin-15 (IL-15) in human rhabdomyosarcoma. osteosarcoma. and Ewing's sarcoma. *Int J Cancer* **71**: 732–736

Meazza R. Gaggero A. Neglia F. Basso S. Sforzini S. Pereno R. Azzarone B and Ferrini S (1997) Expression of two interleukin-15 mRNA isoforms in human tumors does not correlate with secretion: role of different signal peptides. *Eur J Immunol* **27**: 1049–1054

Meng RD. Shih H. Prabhu NS. George DL and El-Deiry WS (1998) Bypass of abnormal MDM2 inhibition of p53-dependent growth suppression. *Clin Cancer Res* **4**: 251–259

Onu A. Pohl T. Krause H and Bulfone-Paus S (1997) Regulation of IL-15 secretion via the leader peptide of two IL-15 isoforms. *J Immunol* **158**: 255–262

Pappo AS. Shapiro DN. Crist WM and Maurer HM (1995) Biology and therapy of pediatric rhabdomyosarcoma. *J Clin Oncol* **13**: 2123–2139

Pesch J. Brehm U. Staib C and Grummt F (1996) Repression of interleukin-2 and interleukin-4 promoters by tumor suppressor protein p53. *J Interferon Cytokine Res* **16**: 595–600

Quinn LS. Haugk KL and Grabstein KH (1995) Interleukin-15: a novel anabolic cytokine for skeletal muscle. *Endocrinology* **136**: 3669–3672

Schweigerer L. Neufeld G. Mergia A. Abraham JA. Fiddes JC and Gospodarowicz D (1987) Basic fibroblast growth factor in human rhabdomyosarcoma cells: implications for the proliferation and neovascularization of myoblast-derived tumors. *Proc Natl Acad Sci USA* **84**: 842–846

Shin TH. Paterson AJ and Kudlow JE (1995) p53 stimulates transcription from the human transforming growth factor alpha promoter: a potential growth-stimulatory role for p53. *Mol Cell Biol* **15**: 4694–4701

Soddu S. Blandino G. Citro G. Scardigli R. Piaggio G. Ferber A. Calabretta B and Sacchi A (1994) Wild-type p53 gene expression induces granulocytic differentiation of HL-60 cells. *Blood* **83**: 2230–2237

Tagaya Y. Bamford RN. Defilippis AP and Waldmann TA (1996) IL-15: a pleiotropic cytokine with diverse receptor/signaling pathways whose expression is controlled at multiple levels. *Immunity* **4**: 329–336

Zhang L. Kashanchi F. Zhan Q. Zhan S. Brady JN. Fornace AJ. Seth P and Helman LJ (1996) Regulation of insulin-like growth factor II P3 promoter by p53: a potential mechanism for tumorigenesis. *Cancer Res* **56**: 1367–1373

© Cancer Research Campaign 1998